# Prevalence and anatomical distribution of incidental and actionable findings on CBCT scans for implant planning

**DOI:** 10.1371/journal.pone.0355052

**Published:** 2026-07-30

**Authors:** Shrey Pandya, Swarna Yerebairapura Math, Justin Kim, Adnan Shah, Saranjeev Lalh, Camila Pacheco-Pereira

**Affiliations:** 1 Mike Petryk School of Dentistry, University of Alberta, Alberta, Canada; 2 Private Practice, Edmonton, Alberta, Canada; 3 Dr. Gerald Niznick College of Dentistry, University of Manitoba, Winnipeg, Canada; Ohio State University, UNITED STATES OF AMERICA

## Abstract

**Objectives:**

To determine the prevalence and anatomical distribution of incidental (IF) and actionable findings (AF) on cone-beam computed tomography (CBCT) scans obtained for dental implant planning, and to evaluate their associations with patient demographics and imaging parameters.

**Materials and Methods:**

This retrospective observational study included all consecutive CBCT scans obtained for implant planning (n = 368). A structured, zone-based evaluation protocol was applied, systematically assessing four anatomical regions: (1) cranium and paranasal sinuses, (2) zygomaticomaxillary/orbital complex including the temporomandibular joint, (3) maxillomandibular region, and (4) structures inferior to the mandible. IF were defined as findings unrelated to the implant planning site, and AF were classified based on the oral and maxillofacial radiologist recommendations for further evaluation, referral, or management rather than confirmed clinical outcomes. Associations between IF, AF, CBCT field of view (FOV), age, and sex were analyzed using univariate and multivariable methods.

**Results:**

IF were observed in 257 patients (69.8%) and AF in 199 (54.1%). IF prevalence increased from 50% in patients ≤40 years to 78.8% in those >70 years, and was higher in males than females (76.2% vs. 64.5%; *p* = 0.01). In univariate analyses, AF were not significantly associated with sex (p = .97) or age (p = .25). However, patients >70 years had higher odds of AF in multivariable analysis, although the overall model was not statistically significant. Zone 2 findings were more common in females than males (21.5% vs. 7.1%; *p* < 0.001). IF were most common in medium FOV scans (72.2%), whereas AF did not vary by FOV (*p* = .94). The most prevalent findings involved the maxillary sinus (35.1%), cervical spine (27.4%), tonsils (15.8%), TMJ (11.7%), and vasculature (8.4%).

**Conclusions:**

IF and findings classified as AF based on OMR recommendations were highly prevalent in CBCT scans obtained for implant planning. Approximately half of the scans presented AF (54%). These findings were distributed across multiple anatomical zones and were associated with age and sex, while AF showed no association with FOV.

## Introduction

The widespread integration of cone-beam computed tomography (CBCT) across dental specialties, including implantology, oral surgery, and periodontology, has significantly improved treatment outcomes [1,2]. Its multiplanar imaging enables three-dimensional (3D) assessment of maxillofacial structures and supports implant planning, now considered the standard of care [1–4], with computer-assisted implant surgery further enhancing the precision of prosthetically driven placement [1,5–11].

Dental education increasingly emphasizes the advantages of CBCT alongside the clinician’s responsibility to recognize radiographic findings beyond the primary area of concern [1,5,7,9,12–14]. However, variability in training and experience may lead to misinterpretation of normal anatomical variants, anomalies, or imaging artifacts as pathology, potentially causing unnecessary patient anxiety and expensive follow-up investigations [15]. Systematic interpretation protocols and specialist reporting, therefore, remain important components of CBCT-based assessment, particularly in academic imaging environments [12–14].

With the expanding use of CBCT in implant dentistry, findings unrelated to the primary diagnostic indication are increasingly being identified [15,16]. The literature defines incidental findings (IF) as findings unrelated to the area of concern and that do not require additional management recommendations, while actionable findings (AF) represent findings requiring further management [13]. Although such detection may improve patient care by identifying otherwise unrecognized conditions, it also raises ethical and legal responsibilities for clinicians [13,15,16]. IF prevalence varies considerably depending on scan indication, field of view (FOV), and classification methodology [17], and full-volume analyses highlight both the frequency of extra-regional findings and the need for standardized interpretation [18].

Despite the growing use of CBCT in implantology, gaps remain regarding the prevalence and distribution of findings beyond the primary implant site, particularly in academic settings [1,19,20]. Existing studies show substantial heterogeneity in populations, imaging protocols, and definitions of incidental and clinically significant findings, limiting cross-study comparisons [16–18], and few have distinguished radiographically detected findings from those considered actionable based on specialist recommendations [13,16–18]. This distinction may improve clinical interpretability by separating findings that warrant further evaluation from those that do not, while acknowledging that report-based recommendations do not necessarily reflect confirmed clinical outcomes. Furthermore, inconsistent evaluation of demographic and imaging-related variables, such as age, sex, and FOV, restricts understanding of their associations with these findings [16–18]. Although recent implant-focused CBCT studies highlight the importance of IF [21], evidence from academic imaging settings remains limited [22].

This study, therefore, aimed to determine the prevalence and anatomical distribution of IF and AF identified on CBCT scans for implant planning at an academic dental imaging center, and to examine their associations with patient demographic and imaging variables. In addition, the study characterized findings classified as actionable based on specialist recommendations.

## Materials and methods

This retrospective observational cross-sectional study was approved by the Institutional Research Ethics Board at the University of Alberta (Pro00120261) and conducted in accordance with the principles of the Declaration of Helsinki [23]. This study was reported in accordance with the Strengthening the Reporting of Observational Studies in Epidemiology (STROBE) guidelines. No additional radiographic imaging was obtained for research purposes. All data were fully anonymized, and no identifiable patient information was available to the investigators. Given the retrospective nature of the study and the use of anonymized records, the requirement for informed consent was waived by the Institutional Research Ethics Board. Anonymized data were accessed for research purposes.

### Study setting and population

All adult patients (≥18 years) referred to the Advanced Imaging Centre, Mike Petryk School of Dentistry, University of Alberta, for CBCT imaging for dental implant planning from July 2021 to July 2024 were considered eligible. As this was a single academic imaging centre, the study population may reflect local referral patterns, institutional imaging protocols, and specialist reporting practices.

Scans were included if they were obtained for implant planning and had a complete radiology report interpreted as part of routine care by an oral and maxillofacial radiologist (OMR); scans without OMR reports, incomplete datasets/reports, or duplicate scans from the same patient during the study period were excluded.

### Sample size

As this retrospective study included all consecutive eligible CBCT scans obtained during the study period, no formal sample size determination was used to guide recruitment. Instead, sample size adequacy was evaluated using an a priori calculation based on prevalence estimates from prior literature. The required sample size was calculated using the formula *n = (Z² × p × (1 − p)) / d²*, assuming a prevalence (p) of 39.7% reported in previous studies, a 95% confidence level (Z = 1.96), and a margin of error (d) of 5%, resulting in a minimum required sample size of 368 [13]. A target sample of 368 was planned to meet this requirement. Sample adequacy was further assessed based on the precision of prevalence estimates, defined by the width of the 95% confidence intervals. This calculation was intended to support estimation of overall prevalence and was not specifically powered to detect subgroup differences across sex, age categories, FOV groups, or anatomical zones.

### CBCT acquisition protocol

CBCT imaging was acquired using iCat Classic and Planmeca Viso G7 systems under standardized centre protocols by the same technician. Imaging parameters, including FOV selection, voxel size, and exposure settings, were determined according to routine clinical implant-planning requirements and manufacturer-recommended acquisition protocols. Acquisition parameters varied according to clinical indication and were not consistently available for retrospective extraction. FOV was selected according to the implant-planning requisition and categorized as small (one arch or less), medium (both arches), or large (coverage extending beyond both arches to include additional craniofacial structures) [13]. Because imaging acquisition reflected routine clinical care, some variability in imaging parameters and anatomical coverage was expected across scans.

### Radiographic assessment and data extraction

Radiographic findings were extracted from OMR reports into a standardized spreadsheet by two independent reviewers (a dentist with oral and maxillofacial surgery training (S.P.) and a final-year DDS student (J.K.). To enhance data consistency, both reviewers followed a predefined data extraction protocol. A subset of scans was jointly reviewed before formal data collection to ensure reviewer calibration and consistency in data interpretation. Discrepancies during extraction were resolved through discussion and consensus. Because radiographic interpretations were derived from finalized OMR reports rather than independent image review, reliability assessment was limited to data extraction consistency rather than diagnostic interpretation.

Extracted data included patient age and sex, implant site(s) and referring department (undergraduate clinic, graduate clinic, or general practice residency (GPR) clinic), FOV category, and CBCT acquisition date. Recorded radiographic variables included alveolar bone quality, condylar and temporomandibular joint (TMJ) abnormalities, airway abnormalities, maxillary sinus abnormalities, and the presence of maxillary sinuses/antral pathologies. Additional findings included periodontal conditions, soft tissue calcifications, ligament ossification, salivary gland abnormalities, cervical spine changes, vascular and other calcifications, and any management or referral recommendations documented in the OMR report.

### Classification of radiographic findings

CBCT volumes were reviewed using a structured, whole-volume evaluation approach designed to ensure systematic assessment beyond the primary implant site. Each scan was assessed by the OMR following a predefined step wise protocol, including multiplanar (axial, coronal, and sagittal) reconstruction review, followed by sequential assessment of each anatomical zone to minimize omission of findings. Radiographic findings were recorded when features deviated from normal anatomical appearance. Operational classification criteria were based on the OMR report, with categorization guided by documented recommendations for follow-up, referral, or management.

In this study, IF were defined as findings unrelated to the primary implant site that did not require additional diagnostic evaluation or management recommendation. AF were defined as findings associated with documented OMR recommendations for further diagnostic work-up, referral, clinical monitoring, or management. For example, sinus mucosal thickening, mucous retention cysts, tonsilloliths, TMJ degenerative changes, and cervical spine degenerative findings were classified as AF only when the OMR report included a recommendation for further evaluation, referral, monitoring, or management; otherwise, they were classified as IF. Classification was therefore based on report-documented specialist recommendations rather than confirmed downstream clinical outcomes.

**Fig 1:**
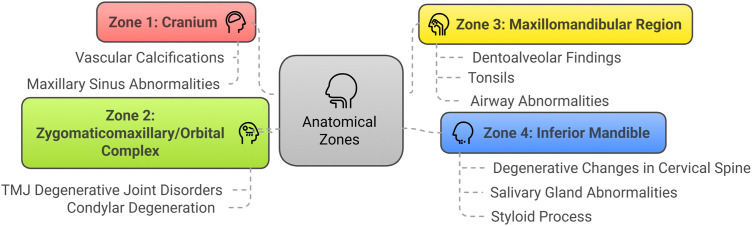
Anatomical zones.

As illustrated in Fig 1, all radiographic findings were classified into four anatomical zones adapted from validated methodologies [13,22]: *Zone 1,* the cranium and paranasal sinuses, including internal carotid artery (ICA), as well as maxillary sinus abnormalities; *Zone 2,* the zygomaticomaxillary and orbital complex, including TMJ degenerative joint disorders (DJD) and condylar degeneration; *Zone 3,* the maxillomandibular region, including dentoalveolar findings, tonsils, and airway abnormalities; and *Zone 4* included structures inferior to the mandible, such as degenerative changes in the cervical spine, salivary gland abnormalities, and the styloid process.

### Statistical analysis

Descriptive statistics were calculated to summarize patient demographics and radiographic findings. Continuous variables (age) were summarized using means, standard deviations, and ranges, while categorical variables (sex and radiographic findings) were summarized using frequencies and percentages. The prevalence of IF and AF was calculated as a proportion of the total study population. Inferential analyses using Chi-square tests were performed to evaluate associations between IF and AF and demographic variables (age and sex), as well as imaging parameters such as CBCT FOV. These analyses were exploratory in nature and intended to identify potential associations across demographic, anatomical, and imaging-related variables. Given the number of subgroup comparisons performed, no formal adjustment for multiple testing was applied; therefore, statistically significant findings, particularly those with marginal p-values, were interpreted cautiously. Multivariable logistic regression analyses were additionally performed to assess the independent association of age, sex, and CBCT FOV on the presence of IF and AF. Results were reported as odds ratios (OR) and 95% confidence intervals (CI). A *p*-value <0.05 was considered statistically significant. All analyses were performed using the Statistical Package for the Social Sciences (SPSS), version 23 (IBM Corp., Armonk, NY, USA).

## Results

**Fig 2:**
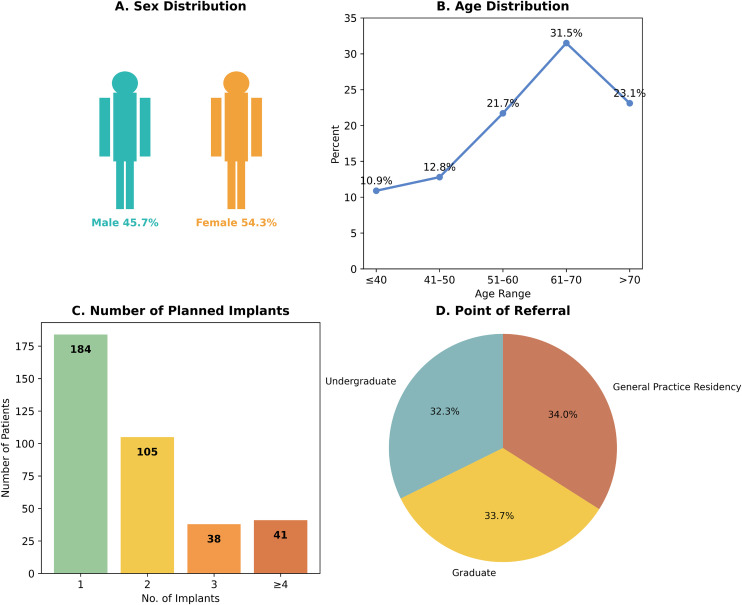
Demographic characteristics of the collected data.

A total of 706 implant sites were identified across 368 CBCT scans obtained for implant planning purposes. The study population included 168 males (45.7%) and 200 females (54.3%), with ages ranging from 18 to 92 years. Overall, radiographic findings were common across the cohort, with several findings demonstrating prevalence in regions beyond the primary implant site, indicating the presence of extra-regional findings on CBCT scans. The descriptive analysis of radiographic findings is presented in Table 1 and S2-S4 tables. The demographic characteristics are illustrated in Fig 2.

**Table 1:** Descriptive summary of radiographic findings identified on CBCT scans (N = 368).

Radiographic finding	Number of patients (N)	%
**Trabecular bone pattern**		
Normal	273	74.2
Sclerotic	8	2.2
Grafted site	34	9.2
Atrophic	53	14.4
**Temporomandibular joint findings**		
Condylar degenerative changes	34	9.2
TMJ degenerative joint disease (DJD)	43	11.7
**Airway and tonsillar findings**		
Tonsilloliths	58	15.8
Airway abnormalities	16	4.3
Sinonasal findings		
Sinusitis-related changes	60	16.3
Mucous retention pseudocyst	69	18.8
**Other findings**		
Periodontal findings	5	1.4
Cervical spine degenerative changes	101	27.4
Vascular calcifications (ICA)	31	8.4
Elongated styloid process	1	0.3
Salivary gland abnormalities	1	0.3

Note: Percentages are calculated based on the total number of CBCT scans reviewed. Patients may present with more than one finding.

### Referral sources, imaging parameters, and anatomical distribution of IF and AF

Referral sources were similarly distributed among the undergraduate implant clinic (32.3%), the periodontology graduate program (33.7%), and the GPR clinic (34.0%). Of the CBCT scans reviewed, 2.2% (N = 8) were acquired using a small FOV, the majority were obtained using a medium FOV (60.6%, N = 223), and the remaining scans were acquired using a large FOV (37.2%, N = 137). Given the limited number of small-FOV scans, comparisons across FOV categories should be interpreted cautiously.

IF were observed in 257 (69.8%) of 368 patients, representing the majority of the study population. As shown in Table 2, IF were distributed across four anatomical zones: Zone 1 (22.8%), Zone 2 (14.9%), Zone 3 (42.4%), and Zone 4 (28.0%), with the highest proportion in Zone 3. AF were identified in 199 patients (54.1%) and were also distributed across anatomical zones, with the highest proportion observed in Zone 3 (32.1%) (Table 2). In contrast, Zone 4 demonstrated a relatively high prevalence of IF (28.0%) but contributed very few AF (0.5%), suggesting that many findings inferior to the mandible were incidental in nature and infrequently associated with additional management recommendations.

**Table 2:** Distribution of IF and AF across anatomical zones.

Radiographic Findings		Number (N)	Percentage (%)
**Incidental findings (IF)**	Zone 1	84	22.8
	Zone 2	55	14.9
	Zone 3	156	42.4
	Zone 4	103	28.0
	**Overall**	**257**	**69.8**
**Actionable Findings (AF)**	Zone 1	84	22.8
	Zone 2	55	14.9
	Zone 3	118	32.1
	Zone 4	2	0.5
	**Overall**	**199**	**54.1**

### Sex-based associations of IF and AF

As shown in Table 3, a statistically significant association was observed between IF and sex (*p*=0.015), with IF identified in 76.2% of males and 64.5% of females. Zone-specific analysis demonstrated a significant difference in Zone 2 IF (*p*<0.001), with a higher prevalence in females (21.5%) compared to males (7.1%). No significant sex-based differences were observed in Zone 1 (*p*=0.680), Zone 3 (*p*=0.423), or Zone 4 (*p*=0.104). These findings suggest that the observed sex-related differences were predominantly localized to the zygomaticomaxillary/orbital complex and TMJ region rather than uniformly distributed across all anatomical zones.

**Table 3:** Association of incidental (IF) and actionable findings (AF) with biological sex.

Radiographic Findings		Male (N = 168)		Female (N = 200)		*p* value
		N	%	N	%	
**Incidental findings (IF)**	**Overall**	**128**	**76.2**	**129**	**64.5**	.015*
	Zone 1	40	23.8	44	22.0	.68
	Zone 2	12	7.1	43	21.5	<0.001*
	Zone 3	75	44.6	81	40.5	.42
	Zone 4	54	32.1	49	24.5	.10
**Actionable findings (AF)**	**Overall**	**91**	**54.2**	**108**	**54.0**	**.97**
	Zone 1	40	23.8	44	22.0	.68
	Zone 2	12	7.1	43	21.5	<0.001*
	Zone 3	55	32.7	63	31.5	.80
	Zone 4	2	1.2	0	0	.12

* *p* < 0.05 Note: Percentages are calculated within each sex group. Patients may present with findings in more than one anatomical zone.

**Fig 3:**
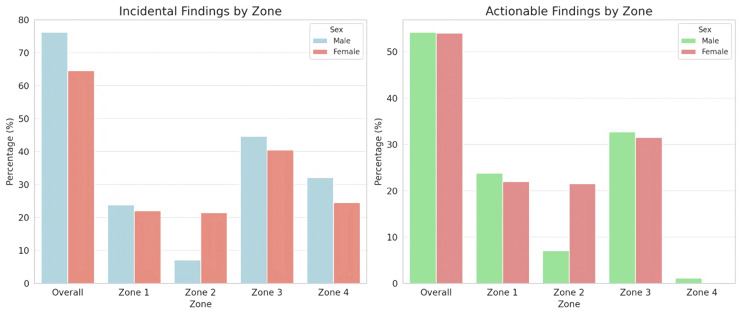
Distribution of IF and AF across anatomical zones by sex.

For AF, no significant difference was observed in overall prevalence between males (54.2%) and females (54.0%) (*p*=0.975). However, Zone 2 AF demonstrated a statistically significant association with sex (*p*<0.001), with a higher prevalence in females (21.5%) compared to males (7.1%). No significant sex-based differences were observed in Zone 1 (*p*=0.680), Zone 3 (*p*=0.800), or Zone 4 (*p*=0.122). Fig 3 illustrates the distribution of IF and AF across the anatomical zones by sex.

### Age-related Associations of IF and AF

As presented in Table 4, IF were significantly associated with age (*p* = 0.012), with prevalence increasing from 50% in patients aged ≤40 to 78.8% in those >70 years old. Anatomical zone-specific analysis demonstrated a significant association between age and Zone 1 (*p* = 0.043), Zone 2 (*p* = 0.035), and Zone 4 findings (*p* < 0.001). In Zone 4, IF were identified in 103 patients, with the highest prevalence observed in those over 70 years (41.2%), and no IF in patients aged  ≤ 40 years. In Zone 1, IF prevalence increased from 10% to 31.8% across the same age groups. In Zone 2, IF increased from 7.5% to 23.5%. No significant association was observed between age and Zone 3 *(p* = 0.430). Many of the age-associated findings identified in Zones 1 and 4 represented degenerative or calcific changes frequently observed in older populations.

**Table 4:** Association of IF and AF with Age.

Radiographic Findings		Age (Years)										*p* value
		<=40 (n = 40)		41-50 (n = 47)		51-60 (n = 80)		61-70 (n = 116)		>70 (n = 85)		
		N	%	N	%	N	%	N	%	N	%	
**Incidental findings (IF)**	Overall	20	50.0	29	61.7	56	70.0	85	73.3	67	78.8	.012*
	Zone 1	4	10.0	9	19.1	14	17.5	30	25.9	27	31.8	.043*
	Zone 2	3	7.5	7	14.9	6	7.5	19	16.4	20	23.5	.035*
	Zone 3	18	45.0	15	31.9	37	46.3	46	39.7	40	47.1	.43
	Zone 4	0	0	6	12.8	19	23.8	43	37.1	35	41.2	<0.001*
**Actionable findings (AF)**	Overall	16	40.0	24	51.1	42	52.5	65	56.0	52	61.2	.25
	Zone 1	4	10.0	9	19.1	14	17.5	30	25.9	27	31.8	.043*
	Zone 2	3	7.5	7	14.9	6	7.5	19	16.4	20	23.5	.035*
	Zone 3	14	35.0	11	23.4	28	35.0	36	31.0	29	34.1	.68
	Zone 4	0	0	1	2.1	0	0	0	0	1	1.2	.39

* *p* < 0.05

AF did not demonstrate a statistically significant overall association with age (*p* = 0.253), although prevalence increased from 40.0% in patients ≤40 years to 61.2% in those >70 years. Zone-specific analysis demonstrated significant associations between age and Zone 1 (*p* = 0.043) and Zone 2 (*p* = 0.035), with AF increasing from 10.0% to 31.8% and from 7.5% to 23.5%, respectively. No significant associations were observed for Zone 3 (*p* = 0.678) or Zone 4 (*p* = 0.392). These findings indicate that although AF prevalence increased numerically with age, statistically significant associations were primarily localized to specific anatomical regions rather than observed uniformly across all zones.

### Association of IF and AF with CBCT FOV

The percentage of scans with IF or AF was calculated for each CBCT FOV group, as shown in Table 5. IF were most prevalent in medium FOV scans (72.2%), followed by large FOV scans (67.2%) and small FOV scans (50.0%); however, these differences were not statistically significant (*p* = 0.28). Similarly, no statistically significant differences in AF were observed across FOV categories (*p* = 0.94). Interpretation of FOV-related analyses should be performed cautiously due to the substantial imbalance in group sizes, particularly the limited number of small-FOV scans, which may have reduced statistical power to detect differences related to anatomical coverage and extra-regional findings.

**Table 5:** Association of IF and AF with CBCT FOV.

Findings		CBCT FOV						*p* -value
		Small (n = 8)		Medium (n = 223)		Large (n = 137)		
		N	%	N	%	N	%	
**Incidental findings**	**Overall**	**4**	**50.0**	**161**	**72.2**	**92**	**67.2**	**.28**
	Zone 1	2	25.0	51	22.9	31	22.6	.99
	Zone 2	0	0	34	15.2	21	15.3	.49
	Zone 3	3	37.5	104	46.6	49	35.8	.12
	Zone 4	1	12.5	69	30.9	33	24.1	.23
**Actionable findings**	**Overall**	**4**	**50.0**	**122**	**54.7**	**73**	**53.3**	**.94**
	Zone 1	2	25.0	51	22.9	31	22.6	.99
	Zone 2	0	0	34	15.2	21	15.3	.49
	Zone 3	2	25.0	76	34.1	40	29.2	.57
	Zone 4	0	0	2	0.9	0	0	.52

* *p* < 0.05

Multivariable logistic regression analysis was performed to evaluate the effects of sex, age, and CBCT FOV on the presence of IF and AF. For IF, the overall model was statistically significant (*p* = 0.007) as described in Table 6. Increasing age was associated with higher odds of IF, with patients aged 61–70 years (OR = 2.62; 95% CI: 1.23–5.58; *p* = 0.012) and >70 years (OR = 3.41; 95% CI: 1.51–7.74; *p* = 0.003) demonstrating significantly greater likelihood compared to those ≤40 years. Female sex was associated with lower odds of IF (OR = 0.59; 95% CI: 0.37–0.95; *p* = 0.029), while CBCT FOV was not significantly associated with IF. However, FOV-related estimates demonstrated wide confidence intervals, likely reflecting the small number of scans within the small-FOV reference category.

**Table 6:** Logistic Regression for Incidental Findings.

Variable		Adjusted OR	95% CI	*P* value
**Sex**	Male	Reference	–	–
	Female	.59	.37−.95	.03*
**Age group**	<=40	Reference	–	–
	41-50	1.59	.67-3.76	.29
	51-60	2.21	.99-4.89	.05
	61-70	2.62	1.23-5.58	.01*
	>70	3.41	1.50-7.74	.003*
**CBCT FOV**	Small	Reference	–	–
	Medium	2.57	.59-11.04	.21
	Large	2.02	.46-8.79	.35

*p < 0.05; Significant

In contrast, the model for AF was not statistically significant (*p* = 0.608), indicating limited predictive value of the included variables, as shown in Table 7. However, patients aged >70 years had higher odds of AF (OR = 2.36; 95% CI: 1.09–5.11; *p* = 0.029), while sex and FOV were not significant predictors. Given the absence of overall model significance, this isolated age association should be interpreted cautiously. Because the small-FOV category included a limited number of scans, FOV-related estimates were interpreted cautiously due to the potential for unstable OR estimates and wide confidence intervals.

**Table 7:** Logistic Regression for Actionable Findings.

Variable		Adjusted OR	95% CI	*P* value
Sex	Male	Reference	–	–
	Female	1.032	0.68-1.57	0.88
Age group	<=40	Reference	–	–
	41-50	1.569	0.67-3.68	.30
	51-60	1.673	0.77-3.63	.19
	61-70	1.926	0.92-4.02	.08
	>70	2.362	1.09-5.11	.03
CBCT FOV	Small	Reference	–	–
	Medium	1.124	0.27-4.68	.87
	Large	1.09	0.26-4.58	.90

*p < 0.05; Significant

## Discussion

Our findings demonstrate a high prevalence of IF (69.8%) and AF (54.1%) on CBCT scans obtained for implant planning. These results are consistent with systematic reviews and full-volume CBCT studies reporting frequent findings beyond the primary area of interest [17,18]. Differences in prevalence across studies likely reflect variation in scan indications, FOV, reporting methodology, and classification criteria [16–18]. Importantly, AF in this study was classified according to OMR recommendations for further evaluation, referral, or management, and does not represent confirmed clinical outcomes. Collectively, these observations highlight the critical role of CBCT in identifying anatomical variations and clinically relevant alterations beyond the primary implant site, supporting its integration into routine diagnostic protocols in both academic and private practice settings [1,5,9,12,13].

One critical and frequently underappreciated aspect of 3D imaging is the ethical, professional, clinical, and legal responsibilities associated with the recognition and management of radiographic findings outside the area of interest, particularly when such findings are incidental or diagnostically indeterminate [15]. In the present study, more than half of all CBCT scans had AF, indicating that findings requiring further evaluation are commonly encountered in routine implant-planning imaging. As CBCT use continues to expand across dental practice, the detection of IF and AF has become increasingly frequent, placing greater responsibility on clinicians to address findings beyond the initial diagnostic intent [15]. The expanding use of CBCT is also reflected in the projected growth of the global CBCT market, expected to reach USD 798.64 million by 2030 [24]. This trend is further amplified by the growing adoption of CBCT in both academic and private practice settings, alongside advancements in imaging technology and increased medico-legal awareness [15].

This study also identified sex-based differences in the prevalence and distribution of IF on CBCT imaging. Although overall IF prevalence was higher in males, the observed sex association was not uniform across anatomical regions. The most prominent differences were localized in Zone 2, which included the zygomaticomaxillary/orbital complex and TMJ-related findings, where females demonstrated approximately threefold greater prevalence than males. This pattern is consistent with previous reports describing a higher susceptibility to TMJ disorders among females, potentially influenced by hormonal, biomechanical, and behavioral factors [7,15]. These findings suggest that sex-related differences may be region-specific rather than generalized across the craniofacial complex. Similar sex-related variation has also been reported for intracranial calcifications, which appear more frequently in women [7].

The present study also demonstrated a significant association between age and the prevalence of IF. The observed increase in findings with advancing age may reflect the cumulative effect of degenerative, inflammatory, and calcific processes that become more prevalent over time. Age-related findings were more common in anatomical regions beyond the immediate implant site, particularly within superior and inferior craniofacial structures, likely reflecting both the anatomical extent of CBCT coverage and the greater frequency of sinus, cervical spine, and soft-tissue related findings in these regions. These observations are consistent with previous studies reporting increased prevalence of degenerative and calcific changes with advancing age [17,18]. Many findings contributing to the increased prevalence observed in older age groups, particularly within Zone 4, represented degenerative changes that may have limited direct relevance to implant planning. Although patients older than 70 years demonstrated higher odds of AF in multivariable analysis, the overall AF regression model was not statistically significant; therefore, this finding should be interpreted cautiously and not viewed as strong predictive evidence. Collectively, these findings reinforce the importance of systematic whole-volume CBCT interpretation across all age groups rather than reliance on demographic risk profiling alone.

In addition to demographic factors, imaging-related parameters may influence the detection of IF and AF. Another study reported a higher frequency of IF with medium FOV scans, in contrast to existing literature suggesting increased detection of AF with larger FOVs [16,25]. These discrepancies likely reflect differences in anatomical coverage, reporting thresholds, and study design, rather than true differences in disease prevalence. Prior research has shown that reducing the FOV can lower patients radiation exposure, emphasizing the need to balance diagnostic yield with radiation risk [6,26]. In the present study, AF did not differ significantly according to FOV; however, interpretation of this finding should be the result of the imbalance in FOV group sizes, particularly the limited number of small-FOV scans. Therefore, the absence of a statistically significant association should not be interpreted as evidence that FOV does not influence the detection of extra-regional findings.

The high prevalence of AF observed in this cohort highlights the importance of comprehensive whole-volume assessment of CBCT scans in clinical practice. Similar to prior implant-focused and full-volume CBCT studies [17,18,21], our findings demonstrate that findings associated with management recommendations are frequently identified outside the primary implant site, particularly within the cranium/sinonasal and maxillomandibular regions. This distribution pattern suggests that limiting interpretation to the implant site alone may increase the risk of overlooking potentially relevant findings, supporting the growing consensus that CBCT interpretation should extend beyond site-specific evaluation to support comprehensive patient assessment and clinical decision-making [1,9]. However, because clinical follow-up data were unavailable, the present study could not determine whether AF ultimately resulted in referral completion, additional imaging, treatment modification, or improved patient outcomes. From a workflow perspective, the frequency and distribution of AF observed in this study suggest the potential value of systematic documentation, referral, and management pathways before implant placement.

To support clinicians as an adjunct tool, artificial intelligence (AI) and augmented intelligence approaches have been proposed as a second opinion on the interpretation of maxillofacial imaging [27,28]. AI-assisted tools may assist CBCT interpretation and reduce observer variability; however, AI was not evaluated in this study and should be considered a future research direction [17,28,29].

Collaboration with OMR may improve detection of extra-regional findings and facilitate appropriate follow-up, particularly given the frequency of AF observed in this cohort [18,21,30]. Interdisciplinary collaboration may also support the timely management of findings such as TMJ degenerative changes, airway abnormalities, and condylar erosions, which may require referral to oral medicine specialists and/or oral and maxillofacial surgeons [31]. Establishing structured referral pathways may help standardize clinical responses to CBCT findings, support continuity of care, and reduce medico-legal risks associated with incomplete scan interpretation [19].

### Educational implications

Dental education should prioritize structured training in CBCT interpretation, including diagnosis and referral pathways. The high prevalence of IF and AF identified across multiple anatomical zones supports training approaches that emphasize systematic whole-volume evaluation rather than a region-limited focus [11,19]. However, these educational implications should be interpreted as practice-related considerations derived from the observed prevalence of findings rather than outcomes directly measured within the present study.

### Limitations and Future Directions

This study has limitations that should be considered when interpreting the findings. The retrospective, single-center design limits generalization, as the study population consisted exclusively of patients referred for CBCT imaging for implant planning within an academic setting. Referral patterns, imaging protocols, patient demographics, and reporting practices may differ from those in community-based or private practice settings, potentially influencing the observed prevalence of IF and AF.

A key limitation is the absence of clinical follow-up data. Although AF were classified based on documented OMR recommendations, it was not possible to determine whether these recommendations resulted in referral completion, additional imaging, treatment modification, or confirmed clinical outcomes. Consequently, findings classified as AF should be interpreted within the context of recommendation-based reporting rather than verified patient outcomes.

Another limitation is the absence of a formal interobserver reliability assessment. Although all CBCT reports were interpreted by a single experienced OMR, and data extraction followed a predefined protocol, variability in interpretation across observers could not be evaluated. While this approach reduced interpretive variability within the datasets, it limited assessment of reproducibility and observer agreement. In addition, findings were derived from routine clinical radiology reports rather than standardized re-evaluation of all imaging datasets. Although this reflects real-world clinical practice, prevalence estimates may have been influenced by reporting thresholds, report completeness, and differences in follow-up recommendations. A standardized independent review of all CBCT volumes by multiple calibrated observers would have strengthened methodological reliability and external validity.

Also, the imbalance across FOV categories, particularly the small number of scans in the small-FOV group (N = 8), was another limitation. This reduced subgroup comparisons and statistical power for evaluating FOV-related associations. Therefore, findings should be interpreted cautiously and should not be considered evidence that FOV has no influence on the detection of extra-regional findings.

Future research should include multicenter prospective studies with standardized imaging protocols and balanced FOV representation to improve generalizability. Longitudinal studies incorporating clinical follow-up are needed to determine whether findings classified as actionable ultimately result in referral completion, additional imaging, treatment modification, or meaningful patient outcomes.

Based on this study population, the findings emphasize the importance of systematic whole-volume CBCT interpretation. The high prevalence of IF and AF beyond the primary implant site suggests that radiographic abnormalities may be encountered throughout the scanned volume. Although the present study did not evaluate educational outcomes, diagnostic performance, or patient management, the findings support consideration of structured interpretation protocols, interdisciplinary communication, and comprehensive review of CBCT volumes within routine clinical practice.

## Conclusions

CBCT scans obtained for implant planning revealed a high prevalence of IF beyond the primary implant site (69.8%), while 54.1% contained findings classified as AF based on OMR recommendations for further evaluation, referral, or management. These findings were distributed across multiple anatomical zones, with the majority identified outside the primary area of clinical interest. IF were associated with age and sex, whereas no association was observed between AF and FOV. However, interpretation of FOV-related findings should be performed cautiously. These findings support systematic whole-volume CBCT interpretation during implant-planning assessment.

## Supporting information

S1 Table STROBE checklist for observational studies. (DOCX)

S2 Table Endodontic findings requiring specialist consultation. (DOCX)

S3 Table Impacted teeth with associated pathology. (DOCX)

S4 Table Residual roots identified on CBCT scans. (DOCX)
