# Supplementary material for: Prevalence and anatomical distribution of incidental and actionable findings on CBCT scans for implant planning
Source: PLoS One. 2026 Jul 30;21(7):e0355052. doi: 10.1371/journal.pone.0355052 (PMC13422833; doi:10.1371/journal.pone.0355052)
Supplement: S1 Table — (DOCX) [file pone.0355052.s001.docx]

**S1 Table. STROBE checklist for observational studies.**

| **STROBE item** | **Recommendation** | **Addressed in manuscript** |
| --- | --- | --- |
| **Title and abstract** | Indicate study design and provide balanced summary | Title; Abstract |
| **Background/rationale** | Scientific background and justification | Introduction |
| **Objectives** | Specific objectives and hypotheses | Introduction |
| **Study design** | Key design elements | Materials and Methods |
| **Setting** | Location, setting, dates | Materials and Methods |
| **Participants** | Eligibility criteria and selection | Materials and Methods |
| **Variables** | Definitions of outcomes and predictors | Materials and Methods |
| **Data sources/measurement** | Source of data and assessment methods | Materials and Methods |
| **Bias** | Efforts to address potential bias | Materials and Methods; Discussion |
| **Study size** | Explanation of study size determination | Materials and Methods |
| **Quantitative variables** | Handling and categorization | Materials and Methods |
| **Statistical methods** | All statistical analyses | Statistical Analysis |
| **Participants (results)** | Numbers included in analyses | Results |
| **Descriptive data** | Participant characteristics | Results |
| **Outcome data** | Outcome events and measures | Results |
| **Main results** | Estimates and statistical findings | Results |
| **Other analyses** | Subgroup or exploratory analyses | Results |
| **Key results** | Summary of findings | Discussion |
| **Limitations** | Study limitations and potential bias | Discussion |
| **Interpretation** | Interpretation in context | Discussion |
| **Generalisability** | External validity | Discussion |
| **Ethics approval** | Ethics approval and consent waiver | Ethics Statement |
| **Funding** | Funding sources and role | Funding Statement |
