# Supplementary material for: Prevalence and anatomical distribution of incidental and actionable findings on CBCT scans for implant planning
Source: PLoS One. 2026 Jul 30;21(7):e0355052. doi: 10.1371/journal.pone.0355052 (PMC13422833; doi:10.1371/journal.pone.0355052)
Supplement: S3 Table — (DOCX) [file pone.0355052.s003.docx]

**S3 Table: Impacted teeth with associated pathology**

| Number of impacted teeth per patient | Number of patients (N) | % |
| --- | --- | --- |
| 1 | 13 | 3.5 |
| 2 | 5 | 1.4 |
| 4 | 1 | 0.3 |

Note: Percentages are calculated using the total number of CBCT scans reviewed (N = 368).
