# Supplementary material for: Prevalence and anatomical distribution of incidental and actionable findings on CBCT scans for implant planning
Source: PLoS One. 2026 Jul 30;21(7):e0355052. doi: 10.1371/journal.pone.0355052 (PMC13422833; doi:10.1371/journal.pone.0355052)
Supplement: S4 Table — (DOCX) [file pone.0355052.s004.docx]

**S4 Table: Residual roots identified on CBCT scans**

| Number of residual roots per patient | Number of patients (N) | % |
| --- | --- | --- |
| 1 | 16 | 4.3 |
| 2 | 2 | 0.5 |

Note: Percentages are calculated using the total number of CBCT scans reviewed (N = 368).
